# Point-of-Care Lateral Flow Detection of Viable *Escherichia coli* O157:H7 Using an Improved Propidium Monoazide-Recombinase Polymerase Amplification Method

**DOI:** 10.3390/foods11203207

**Published:** 2022-10-14

**Authors:** Alka Rani, Charles Chinyere Dike, Nitin Mantri, Andrew Ball

**Affiliations:** 1 ARC Training Centre for the Transformation of Australia’s Biosolids Resource, School of Science, RMIT University, Bundoora West, VIC 3083, Australia; 2 The Pangenomics Laboratory, School of Science, RMIT University, Melbourne, VIC 3083, Australia; 3 The UWA Institute of Agriculture, The University of Western Australia, Perth, WA 6009, Australia

**Keywords:** *Escherichia coli* O157:H7, recombinase polymerase amplification, propidium monoazide, food and beverage, viable but non-culturable

## Abstract

The detection of both viable and viable but non-culturable (VBNC) *Escherichia coli* O157:H7 is a crucial part of food safety. Traditional culture-dependent methods are lengthy, expensive, laborious, and unable to detect VBNC. Hence, there is a need to develop a rapid, simple, and cost-effective detection method to differentiate between viable/dead *E. coli* O157:H7 and detect VBNC cells. In this work, recombinase polymerase amplification (RPA) was developed for the detection of viable *E. coli* O157:H7 through integration with propidium monoazide (PMAxx). Initially, two primer sets, targeting two different genes (*rfbE* and *stx*) were selected, and DNA amplification by RPA combined with PMAxx treatment and the lateral flow assay (LFA) was carried out. Subsequently, the *rfbE* gene target was found to be more effective in inhibiting the amplification from dead cells and detecting only viable *E. coli* O157:H7. The assay’s detection limit was found to be 10^2^ CFU/mL for VBNC *E. coli* O157:H7 when applied to spiked commercial beverages including milk, apple juice, and drinking water. pH values from 3 to 11 showed no significant effect on the efficacy of the assay. The PMAxx-RPA-LFA was completed at 39 °C within 40 min. This study introduces a rapid, robust, reliable, and reproducible method for detecting viable bacterial counts. In conclusion, the optimised assay has the potential to be used by the food and beverage industry in quality assurance related to *E. coli* O157:H7.

## 1. Introduction

Food and water-borne diseases caused by microbial pathogens represent a major global threat, accounting for approximately 80% of all gastrointestinal disorders [1,2]. Among the bacterial pathogens, enterohemorrhagic *Escherichia coli* (EHEC) strain O157:H7 represents one of the major foodborne pathogens; the first outbreak of this pathogenic strain was reported in the United States in 1982, associated with the consumption of hamburgers [3]. It poses a serious threat to public health, water, and the food and beverage industry [2,4,5,6,7,8]. Several outbreaks of *E. coli* O157:H7 associated with food consumption have been reported by the Centre for Disease Control and Prevention (CDC); for example, in 2018, a multistate outbreak was reported in the USA linked to romaine lettuce; 210 people fell sick, 96 were hospitalised, 27 developed haemolytic uremic syndrome (HUS) (i.e., permanent renal failure) and 5 people died [9]. *E. coli* O157:H7 can be transmitted through contaminated water and food such as lettuce, tomato, milk, eggs, and meat [2,4,8,10].

The detection of this foodborne pathogen is complicated by its ability to exist in a viable but non-culturable state (VBNC). The VBNC state is a distinctive stage in which cells can remain metabolically active and can be virulent; however, these cells cannot multiply and grow on nutrient media [11]. This can result in false-negative results in the plate counting method [1]. Various environmental stress conditions such as low temperature, osmotic variation, drying, and pH change are responsible for the induction of the VBNC state, also known as the self-protective strategy [12,13]. Several foodborne pathogens are reported to enter the VBNC state including Shiga toxin *E. coli* O157:H7 (STEC), *Listeria monocytogenes*, *Salmonella* sp., and *Campylobacter* sp. [11,14,15,16]. Under favourable environmental conditions, the VBNC cells can become virulent and cause disease [14]. An outbreak caused by the consumption of VBNC *E. coli* O157:H7 contaminated salted salmon roe in Japan provides evidence of the associated risk from VBNC bacteria to public health and safety [17]. Therefore, the detection of VBNC *E. coli* O157:H7 is vital to control the spread of its infection and improve public health and well-being.

To date, the most applied detection methods for *E. coli* O157:H7 in the VBNC state are classical approaches based on a combination of two or more approaches such as the enumeration of culturable cells with differential staining and direct microscopy count using a LIVE/DEAD BacLight Bacterial Viability Kit to identify metabolically active cells, or PCR-based gene target identification combined with a selective growth step [8,14,18]. The LIVE/DEAD BacLight Bacterial Viability Kit uses cell permeant (SYTO9) and cell impermeant (propidium iodide, PI) dyes to interact with live and dead cells, respectively [19]. However, this approach requires growing the bacterium on a selective medium, which needs significant technical expertise and incubation conditions [20]. For an effective outbreak control, early pathogen detection is essential; this prompted research into the field of culture-independent approaches, focusing on nucleic acid amplification methods such as polymerase chain reaction (PCR) and real-time PCR (qPCR) [8,21,22]. Nevertheless, several isothermal DNA amplification methods have recently been developed with fast, simple, and cost-effective benefits [23]. Examples of these methods include loop-mediated isothermal amplification (LAMP), helicase-dependent amplification (HDA), recombinase polymerase amplification (RPA), rolling circle amplification (RCA), strand displacement amplification (SDA), and single primer isothermal amplification (SPIA) [23,24,25,26]. However, the differentiation of viable/dead and VBNC bacteria remains a major shortcoming of all nucleic acid-based detection methods.

As a consequence, nucleic acid amplification based assays have been developed utilising various DNA-intercalating dyes such as ethidium monoazide (EMA) and propidium monoazide (PMA), which enables the differentiation of VBNC and dead cells [27]. Propidium monoazide, which is an analogue of PI, has been successfully applied to the selective detection of live *E. coli* O157:H7 when combined with PCR, real-time PCR, LAMP, recombinase-aided amplification (RAA), and cross priming amplification (CPA) [1,4,10,14,28,29]. Propidium monoazide, a phenanthridine dye with an azide group cross-links irreversibly with organic molecules such as DNA, forming a PMA–DNA complex. The photo-induced DNA complex becomes insoluble and therefore not amplifiable upon exposure to bright visible light. In this way, free DNA released from dead cells is not amplified. In contrast, bacteria with an intact membrane excludes PMA, resulting in the amplification of the extracted DNA [27,28]. A new improved version of PMA, PMAxx has recently been introduced, which shows improved efficacy in terms of binding to the DNA of dead cells [22]. However, the optimisation of the PMAxx assay is important because various factors such as PMAxx concentration, pH of the medium, amplicon size, and bacterial concentration affect the amplification efficiency of the PMAxx pre-treated cells [29,30,31].

Recombinase polymerase amplification (RPA), first reported by Piepenburg et al. [32], has emerged as a promising isothermal DNA amplification method, which has led to significant further research since it was first introduced [32,33]. Recombinase polymerase amplification can be completed with one primer set using a constant temperature ranging from 37–42 °C, with incubation for 5–20 min [33]. Indeed, many studies report the detection of *E. coli* O157:H7 in food and beverage (milk, salad, raw beef, apple juice, and drinking water) using RPA [34,35,36]. However, no attempt has been made so far to develop an RPA-LFA assay aimed at differentiating viable and dead *E. coli* O157:H7 cells. Moreover, to the best of the authors knowledge, there are no reports for the point-of-care detection of VBNC *E. coli* O157:H7 from food and beverages using PMAxx-RPA-LFA. Therefore, the development of RPA technology for the detection of VBNC *E. coli* O157:H7 cells while excluding dead cells represents a significant innovation.

In this study, we aimed to establish, integrate, and optimise RPA-LFA technology with PMAxx dye for the differentiation of live/dead cells as well as the detection of VBNC *E. coli* O157:H7. Specifically, we report on (i) the selection of a specific primer set targeting unique genes (*rfbE* and *stx2*); (ii) optimisation of PMAxx concentration for the detection of only live *E. coli* O157:H7; (iii) assessment of the impact on the PMAxx-RPA-LFA assay of varying ratios of viable:dead cells; (iv) assay validation in commercially available milk, water and apple juice samples; and (v) evaluation of the effect of pH on the PMAxx-RPA-LFA assay.

## 2. Results

### 2.1. Confirmation of Viable and Non-Viable Cells

**Figure 1:**
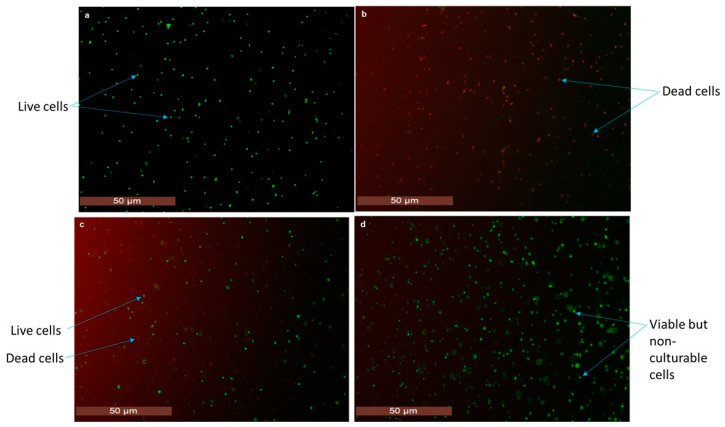
Characterisation of the *E. coli* O157:H7 strain using fluorescence microscopy with the LIVE/DEAD BacLight Viability Kit. (**a**) 100% live bacteria. (**b**) 100% dead bacteria. (**c**) Mixed live:dead (50:50) bacteria. (**d**) VBNC bacteria. Green and red fluorescence represent live and dead cells, respectively.

Dead bacterial cells including the *E. coli* O157:H7 isolate (ATCC 43895) were prepared by heating at 95 °C for 15 min. Cell viability was assessed by plate culturing. Additionally, the viability of the treated *E. coli* O157:H7 was assessed using the LIVE/DEAD BacLight Bacterial Viability Kit (Molecular Probes, Eugene, OR, USA). Under the fluorescent microscope (Leica Microsystems, Hesse, Wetzlar, Germany), live cells (in the exponential state) produced green fluorescence (Figure 1a), confirming the integrity of the bacterial cell membrane, whereas red fluorescence was produced by the dead cells (Figure 1b), confirming the presence of disrupted cells. A mixed population of live and dead cells produce mixed coloured (red and green) fluorescence (Figure 1c) [14].

### 2.2. Optimisation of PMAxx Concentration for the Recombinase Polymerase Amplification Assay (RPA)

**Figure 2:**
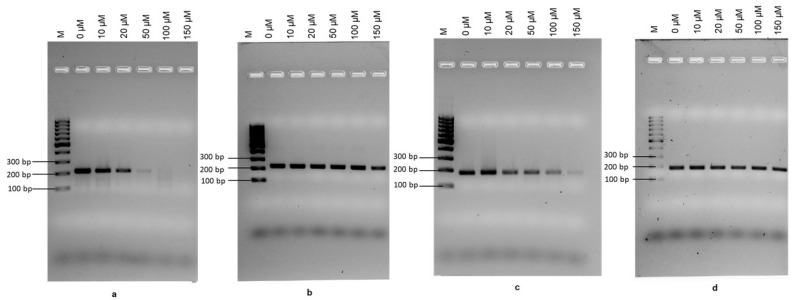
Optimisation of the PMAxx-recombinase polymerase assay (RPA). (**a**–**d**) PMAxx concentrations from 0 to 150 µM; Lane M: molecular marker, Lane 2 to 7: RPA amplified DNA from *E. coli* O157:H7. (**a**,**b**) DNA amplified with the rfbE F1/R1 primer: (**a**) PMAxx treated dead cells, (**b**) PMAxx treated live cells. (**c**,**d**) DNA amplified with stx F1/R1 primer, (**c**) PMAxx treated dead cells, (**d**) PMAxx treated live cells.

The PMAxx concentration required to completely inhibit DNA amplification from dead cells was determined by treating heat-killed *E. coli* O157:H7 cells (2.3 × 10^8^ CFU mL^−1^) with working PMAxx concentrations of 0 µM, 10 µM, 20 µM, 50 µM, 100 µM, and 150 µM. Alongside, 0–150 µM PMAxx concentrations were applied to the same number of viable cells to evaluate the interference of PMAxx on cells with membrane integrity. In this study, *rfbE* and *stx* genes were targeted. An aliquot (100 µM) of PMAxx completely inhibited the amplification of DNA from heat-killed *E. coli* O157:H7 using the *rfbE* primers without any interference in terms of amplifying DNA from viable cells (Figure 2a). However, when the *stx* gene was targeted, the amplification of DNA from dead cells continued across the range of PMAxx concentrations used, although some inhibition was seen in DNA amplification when high PMAxx concentration was used (Figure 2c). In addition, no reduction in amplification was observed when DNA from viable cells was used, irrespective of the gene (Figure 2b,d). The raw data gel images for RPA-EF are provided in Figure S1.

### 2.3. Specificity Assessment

Heat-inactivated cells of 21 bacterial isolates were used to test the specificity of primers (*rfbE* and *stx* genes) for PMAxx-RPA-LFA. No amplification was seen in any dead bacterial isolate with the *rfbE* gene primers; however, when *stx* primers were used, positive amplification was observed in all *E. coli* O157:H7 isolates, suggesting that no DNA inhibition occurred following PMA treatment (Table 1).

### 2.4. Assessment of the Interference of PMAxx to the Recombinase Polymerase Amplification (RPA)

**Figure 3:**
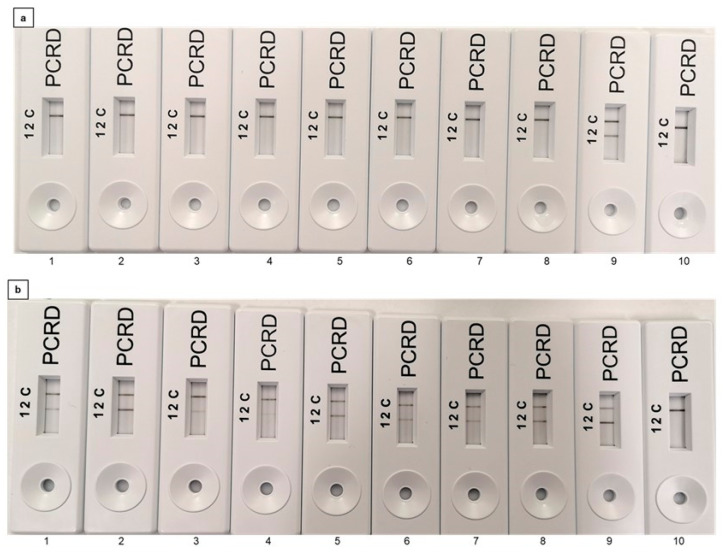
PMAxx-recombinase polymerase assay-lateral flow assay (RPA-LFA) for serially diluted dead *E. coli* O157:H7. (**a**) Targeting the *rfbE* gene; (**b**) targeting the *stx* gene. From left to right (LFA 1–8), the concentrations of dead *E. coli* O157:H7 were 10^8^ CFU mL^−1^, 10^7^ CFU mL^−1^, 10^6^ CFU mL^−1^, 10^5^ CFU mL^−1^, 10^4^ CFU mL^−1^, 10^3^ CFU mL^−1^, 10^2^ CFU mL^−1^, and 10^1^ CFU mL^−1^, LFA 9: live bacterial control at a concentration of 10^8^ CFU mL^−1^ and LFA 10: non-template control (NTC).

The effect of increasing concentrations of dead cells on the PMAxx-RPA method was evaluated. Thermally inactivated dead cells were used in the concentration range of 10^8^ CFU mL^−1^ to 10^1^ CFU mL^−1^ and treated with PMAxx prior to RPA-LFA and RPA-EF detection. No signal amplification was observed in all cell counts using the *rfbE* gene target (Figure 3a and Figure S2a), confirming no interference in the PMAxx-RPA assay; in contrast, signal amplification was seen in all dilutions when the *stx* gene was targeted, confirming the incomplete amplification inhibition in PMAxx pre-treated DNA when the *stx* gene was used (Figure 3b and Figure S2b). On agarose gels, no interference was observed in the amplification of DNA from the live control cells when used at the same cell concentration (Figure S2a,b). An overview of all the results presented in Figure 3 is presented in Table S1a.

In addition, viable and dead *E. coli* O157:H7 at a concentration of 10^8^ CFU mL^−1^ were mixed in different ratios (vol/vol); 0:100, 30:70, 50:50, 70:30, 100:0 and treated with PMAxx (100 µM) prior to RPA-LFA and RPA-EF. No amplification was observed in the 100% dead cells when the *rfbE* primers were used (Figures S3a and S4a). In contrast, reduced DNA amplification was seen in RPA-EF (Figure S4c) with 100% dead cells when the *stx* gene was targeted, indicating interference in the PMAxx-RPA assay. Recombinase polymerase assay-lateral flow assay is more sensitive than RPA-EF; therefore, it produces a strong signal amplification with 100% dead cells with the *stx* gene target (Figures S3c and S4c, Table S1b). This may result in an overestimation of *E. coli* O157:H7 in terms of metabolically active cells. Further validation was carried out by mixing viable:dead cells in proportions of 10^2^:10^8^, 10^3^:10^8^, 10^4^:10^8^, 10^5^:10^8^, and 10^6^:10^8^ prior to PMA treatment. The lowest number of viable cells detected in the assay was determined as the LOD (CFU mL^−1^). Viable cells were amplified in all samples with LOD 10^3^ CFU mL^−1^ and 10^2^ CFU mL^−1^ with RPA-EF and RPA-LFA, respectively. The results in Table S1c confirm that the DNA of mixed (viable:dead) cells was detected, which confirms the detection of *E. coli* O157:H7 using the developed PMAxx-RPA assay (Figures S3b,d and S4b,d).

### 2.5. Validation of the PMAxx-Recombinase Polymerase Amplification (RPA) in Spiked Commercial Samples

Following validation of PMAxx-RPA using pure culture with both primer sets targeting two different genes (*rfbE* and *stx2*), the assay was evaluated using commercial samples (full-cream milk, apple juice, and drinking water) using only the *rfbE* F1/R1 primer set. All of the commercial samples were initially confirmed as negative for the presence of *E. coli* O157:H7 using the *E. coli* O157 latex test in conjugation with SMAC (to confirm the sorbitol non-fermenting colourless colonies) [37]. All samples were spiked with viable and dead *E. coli* O157:H7 at ratios of 10^2^:10^8^, 10^3^:10^8^, 10^4^:10^8^,10^5^:10^8^, 10^6^:10^8^, and 0:10^8^. The results confirm that in the tested samples, the presence of viable *E. coli* O157:H7 down to a concentration of 10^2^ CFU mL^−1^ was detected by PMAxx-RPA-LFA and EF without any significant difference between signal amplification in the presence of dead cells (Figures S5 and S6, Table S2a). Importantly, no signal amplification was seen in the presence of 100% dead *E. coli* O157:H7 in all commercial samples (LFA 13, Figure S5a–c).

### 2.6. Induction of Viable but Non Culturable (VBNC) E. coli O157:H7

**Figure 4:**
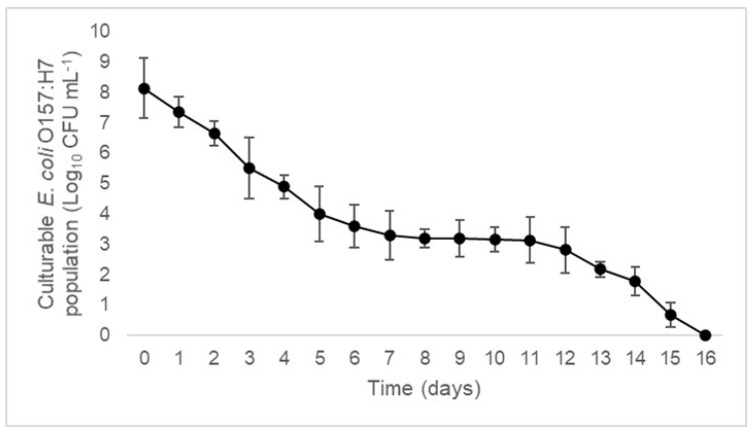
Decline in culturable *E. coli* O157:H7 (CFU mL^−1)^ in 7% NaCl solution at 37 °C over 16 days. (●) represents the mean of the viable count (n = 3) determined by plate counting. Error bars represent the standard deviation of the mean of three replicates.

To investigate the established PMAxx-RPA-LFA assay for real-time practical application on stressed metabolically active cells, *E. coli* O157:H7 cells were induced into the VBNC state by inducing osmotic stress conditions (in 7% NaCl at 37 °C) over 16 days. Throughout the exposure of *E. coli* O157:H7 to osmotic stress, cell counts (CFU mL^−1^) were monitored using plate counting. A gradual reduction in the number of CFU mL^−1^ was observed over time, indicative of the induction of the non-culturable state. A complete reduction in cell count (˂1 CFU mL^−1^) was achieved after 16 days, indicating a non-culturable cell population (Figure 4). Viability of the non-culturable cells was determined by examining the bacterial membrane integrity using the LIVE/DEAD BacLight Bacterial Viability Kit coupled with fluorescence microscopy. The SYTO 9 dye fluoresced all cells as green coccoid cells (Figure 1d) with longpass filter sets, confirming the VBNC *E. coli* O157:H7.

### 2.7. Analysis of Viable but Non Culturable (VBNC) E. coli O157:H7 in Spiked Samples

**Figure 5:**
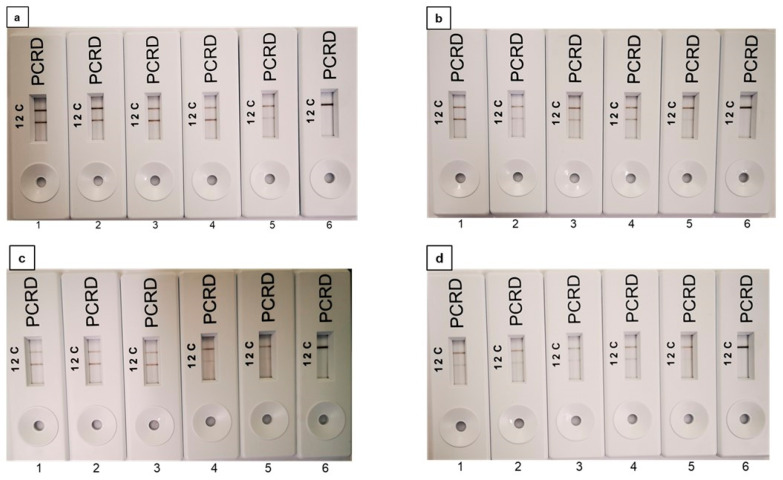
PMAxx-recombinase polymerase assay-lateral flow assay (RPA-LFA) for the detection of VBNC *E. coli* O157:H7 from (**a**) pure culture; (**b**) milk; (**c**) drinking water; (**d**) apple juice. LFA 1 to 6: 9-fold serially diluted VBNC *E. coli* O157:H7 cells with counts ranging from 1.3 × 10^6^ CFU mL^−1^ to 1.3 × 10^2^ CFU mL^−1^, respectively. LFA 6: NTC.

In terms of exploring the possibility of applying the PMAxx-RPA-LFA assay for the detection of VBNC cells in beverages, VBNC *E. coli* O157:H7 was spiked into milk, drinking water, and apple juice at concentrations ranging from 10^2^ to 10^6^ CFU mL^−1^ and subsequently analysed using PMAxx-RPA-LFA. The LOD for VBNC cells in milk, drinking water, and apple juice was found as 10^2^ CFU mL^−1^ including pure *E. coli* O157:H7 (Figure 5a–d). No amplification was seen in the NTC where 0 CFU mL^−1^ VBNC cells were used (LFA 6, Figure 5a–d). The results can be found in Table S2b.

### 2.8. Effect of pH on the Efficiency of Assay

To evaluate the effect of pH on PMAxx-RPA-LFA for *E. coli* O157:H7, water with varying pH (i.e., 3, 5, 7 and 11) was inoculated with 2.3 × 10^8^ CFU mL^−1^ dead cells and treated with PMAxx before RPA-LFA. No DNA amplification was observed at all the tested pH containing dead bacterial cells, confirming that pH did not significantly impact PMAxx binding and inhibition of amplification using the *rfbE* gene target from dead cells (Figure S7).

## 3. Discussion

Food safety represents a key factor in the sustainable growth of the food and beverage industry [38]. Traditionally, the microbial analysis of food has been based on conventional plate count methods combined with PCR; however, advanced diagnostic methods such as qPCR, RPA, and LAMP have been found to be a more appropriate alternative for the rapid and specific detection of foodborne pathogens [1,22,25,26,34,39,40,41]. The presence of *E. coli* O157:H7, an important food and water-borne pathogen can be readily identified and enumerated using nucleic acid amplification-based methods. However, these methods cannot differentiate between dead/viable *E. coli* O157:H7 because the DNA released from dead cells can persist in food samples, resulting in an overestimation of the bacteria [30,42,43]. To overcome these issues, a DNA intercalating dye, PMAxx has been proposed to be combined with nucleic acid amplification methods. PMAxx can enter membrane-compromised cells and covalently bind to cellular DNA by photocatalytic cross-linking, resulting in no DNA amplification [28,29,44,45,46]. The application of PMAxx with qPCR, LAMP, and PCR has been reported to quantify live *E. coli* O157:H7 in various food samples [1,4,14,47,48,49]. However, the application of PMAxx with recombinase polymerase amplification (RPA) has not yet been reported.

The RPA-based assay has been reported for the detection of *E. coli* O157:H7 in food samples, using sets of primers targeting specific genes such as *rfbE, stx1, stx2, eae, uidA,* and *fliC* [29,34,35,50,51,52]. In this present study, two primer sets were selected from the previously tested primers targeting the *rfbE* and *stx2* genes based on their reported sensitivity and specificity [34,35]. Herein, we developed and optimised a point-of-care assay using RPA-LFA technology combined with PMAxx to inhibit DNA amplification from non-viable (dead) cells.

The PMAxx concentration is crucial because low concentrations may not be sufficient to bind the DNA of dead cells, whereas a high concentration can result in an underestimation of live cells due to interference with DNA from viable cells [14,29,53]. Therefore, in this study, the effect of PMAxx concentration on DNA amplification using the RPA-LFA assay was examined. A PMAxx concentration of 100 µM was found to eliminate DNA amplification from non-viable *E. coli* O157:H7, while no inhibition in DNA amplification was seen when viable cells were used. These findings do not concur with those from a previous study [54] that reported the inhibition of DNA amplification in dead *E. coli* O157:H7 cells at 10 µg mL^−1^ PMA using a PCR assay. This may be due to different detection efficiencies associated with different assays. However, our results are in agreement with a report on the detection of live *E. coli* using qPCR and PMA [53]. The optimum PMAxx concentration may vary depending on the ability of the dye to penetrate through the cell membrane of different bacteria [14,55]. In this current study, between the two primer sets, *rfbE* F1/R1 showed the complete inhibition of DNA amplification from dead cells; this result can potentially be explained by the differing size of the two genes as the binding of PMAxx to free-standing DNA is dependent on the size of the amplicon. PMAxx was reported to be more effective at excluding amplification from dead cells when longer amplicons were used compared with when used with smaller amplicons [56]. Amplicons below 200 bp are not recommended with qPCR [57]. The *rfbE* F1/R1 primer set also showed reproducibility and consistency with variable parameters between viable and non-viable cells, showing the robustness of the PMAxx-RPA-LFA assay [30].

The effect of PMAxx concentration was investigated by comparing the DNA amplification by RPA-EF. A PMAxx concentration of 100 µM and a dye binding time of 10 min was optimised for differentiating viable/dead *E. coli* O157:H7 using the *rfbE* F1/R1 primer set. The optimal PMAxx concentration was 10-fold higher than that reported in a previous study, based on a PMA-LAMP assay that worked at 10 µM and the same dye binding time [14]. This discrepancy may be due to the use of different gene targets or different amplicon lengths, as amplicon size has been reported to affect the signal efficiency upon integration with PMA [29,31]. In the present study, different results were observed for the two gene targets (*stx* and *rfbE*) using RPA-LFA, likely due to the difference in the amplicon size of the primers. The *stx2* F1/R1 amplified a 179 bp sequence whereas *rfbE* F1/R1 amplified a 216 bp sequence; according to a previous study, an amplicon size between 200 and 300 bp allowed a significant difference between the live and dead detection values using viable qPCR (v-qPCR). A minimum of 200 bp amplicon length was found to be appropriate for use with V-qPCR; this is also likely to be the case for RPA-LFA [57]. Furthermore, in a recent study, a significant reduction in RPA amplification from dead cells was reported for *Cronobacter sakazakii* from infant formula when long amplicons were used [31].

In the developed PMAxx-RPA-LFA, negative amplification with 21 heat-inactivated bacterial isolates with *rfbE* F1/R1 primers confirmed the binding of PMAxx to free DNA alongside the specificity of the primers [34]. The specificity of primers targeting the *stx* gene agrees with previous reports; however, no DNA inhibition was seen when the live target bacteria was used [34]. It has also been suggested that the presence of high concentrations of non-viable cells reduces the performance of PMA due to the presence of predator cells (metabolically inactive but have cell integrity) [30,58]. However, in the present study, a 100 µM concentration of PMAxx was found to be suitable to detect 2 log_10_ units of viable bacteria mixed with 7 log_10_ of dead *E. coli* O157:H7. pH has also been reported to be an important parameter affecting PMAxx binding [31]. Therefore, this was investigated in the study. However, no difference in RPA amplification was observed at different pH.

The results of fluorescence microscopy confirmed the dead cells (red) in samples where no DNA amplification was observed. The VBNC were visualised as coccoid instead of rod shaped under fluorescence microscopy, as previously reported [14]. This relation between fluorescence microscopy and PMAxx-associated nucleic acid amplification methods has also been previously reported [14,59].

To validate the potential of PMAxx-RPA-LFA for the food and beverage industry, the enumeration of *E. coli* O157:H7 was performed in milk, drinking water, and apple juice. The results obtained confirmed the compatibility of the assay in complex matrices using a mixture of dead/live or VBNC *E. coli* O157:H7. No significant difference was observed in VBNC detection for all three spiked samples, supporting the real-time application of the proposed assay for multiple matrices with reliable reproducibility. Our results support and extend the results from previous studies in determining dead/live or VBNC *E. coli* O157:H7 in various complex matrices combining PMA dye with LAMP, qPCR, and PCR [1,4,10,14,46,54]. Some reports suggest that VBNC cells can reacquire their active state and become viable in the presence of nutrients and favourable conditions, However, no resuscitation of *E. coli* O157:H7 cells was observed in the presented study when VBNC cells were transferred to water, apple juice, and milk [11,15,46]. The revival capacity of different cells may vary with different food matrices or environmental conditions. In this study, for all commercial samples, 2 log_10_ of VBNC cells were detected, which were unable to grow on nutrient media plates. To our knowledge, this is the first study to detect VBNC and differentiate viable/dead *E. coli* O157:H7 from milk, drinking water, and apple juice using the PMAxx-RPA-LFA assay. The developed assay has the potential for use as a point-of-care assay in the food and water sectors to detect only viable or VBNC bacteria for quality assurance purposes if integrated with simple, portable DNA extraction methods such as paper based nucleic acid extraction [60,61]. Additionally, further research aimed at enhancing the sensitivity of the assay may enhance the applicability of the assay for use during outbreaks related to food and water, resulting in improved public health and well-being.

## 4. Material and Methods

### 4.1. Bacterial Isolates and Culture Conditions

In this study, 13 isolates of *E. coli* and other bacterial species including *Staphylococcus aureus*, *Bacillus cereus*, *Klebsiella pneumonia*, *Pseudomonas aeruginosa*, *Shigella dysenteriae*, *Shigella flexneri*, *Salmonella enteritidis*, and *Listeria monocytogenes* were used (Table 1). Cultures were obtained from the RMIT microbial culture collection (RMIT University, Bundoora, Victoria, Australia) and subcultured on nutrient agar (NA) (Signa-Aldrich, Burlington, MA, USA). *E. coli* O157:H7 isolates were confirmed after plating on Sorbitol MacConkey agar (SMAC) (Sigma-Aldrich, Burlington, MA, USA) and using the *E. coli* O157 Latex Test Kit (Thermo Fisher Scientific, Waltham, MA, USA). *E. coli* O157:H7 ATCC 43895 was used as a positive target. Pure cultures of all isolates were inoculated into nutrient broth (NB) with overnight incubation at 37 °C with shaking (180 rpm). Growth was monitored by measuring the optical density at 600 nm (OD_600_) and by traditional plate counting (CFU mL^−1^) using NA (Sigma-Aldrich, Burlington, MA, USA).

### 4.2. Preparation of Non-Viable Cells

For the preparation of dead (non-viable) cells, a rapid thermal lysis method was selected (Nocker et al. 2006). An aliquot (1 mL) of bacterial cell suspension containing 1 × 10^8^ CFU mL^−1^ was centrifuged at 5000× *g* for 10 min. The supernatant was removed, and the pellet resuspended in 1X PBS (100 µL). Resuspended cells were incubated at 95 °C for 15 min with agitation in a thermomixer (Eppendorf, Eppendorf, Hamburg, Germany). Following heat treatment, samples were taken for PMAxx treatment, as described in Section 4.3.

For confirmation of live and dead *E. coli* O157:H7, plate counting was used. The presence of live and dead cells was further confirmed using a LIVE/DEAD BacLight Bacterial Viability Kit (Molecular Probes, Eugene, OR, USA) according to the manufacturer’s guidelines. Briefly, *E. coli* O157:H7 (500 µL) was mixed with 1.5 µL of dyes SYTO 9 and propidium iodide (PI) (1:1 vol/vol) and incubated in the dark for 25–30 min. Two microliters of stained cells were added to the slide, covered with a coverslip, and examined by fluorescence microscopy using a Leica DM2500 (Leica Microsystems, Wetzlar, Hesse, Germany) with GFP (for green fluorescence) and TX (for red fluorescence) longpass filter cubes. Overlay images were acquired for the brightness, white balance, exposure, and gain adjustments to obtain the best contrast between green (live) and red (dead) cells

### 4.3. Propidium Monoazide Optimisation and DNA Extraction

In this study, propidium monoazide (PMAxx, Biotium, Inc., Hayward, CA, USA) at a 20 mM concentration in water was used as a stock solution. For PMAxx optimisation, 1 mL of both viable and dead cells (2.3 × 10^8^ CFU mL^−1^) were centrifuged at 5000× *g* for 10 min and the pellet resuspended in 1X PBS (100 µL). Both the viable and dead cell suspensions were separately treated with different concentrations of PMAxx (0 µM, 10 µM, 20 µM, 50 µM, 100 µM, and 150 µM). PMAxx dye was added to all the tested cells (dead or viable) and incubated in the dark for 10 min with agitation at 120 rpm. Tubes were then transferred to PMA Lite (Biotium, Hayward, CA, USA), a LED photolysis device for 15 min followed by 10 min of incubation on ice. DNA was then extracted from all PMAxx treated samples using the Quick-DNA Faecal/Soil Microbe Kit (Zymo Research, Irvine, CA, USA) as per the manufacturer’s guidelines. Extracted DNA from all samples were quantified using a Nanodrop (Thermo Fisher Scientific, Waltham, Massachusetts, USA) and stored at −20 °C until use in the recombinase polymerase amplification-electrophoresis (RPA-EF) assay.

To observe the effect of the optimised PMAxx concentration on variable numbers of dead cells, 10-fold serially diluted *E. coli* O157:H7 cells ranging from 10^8^ to 10^1^ CFU mL^−1^ were heat inactivated and mixed with 100 µM PMAxx; DNA was then extracted. Live cells were included for comparison using the same concentrations as that of the dead cells.

### 4.4. Propidium Monoazide-Recombinase Polymerase Assay (PMAxx-RPA Assay)

**Figure 6:**
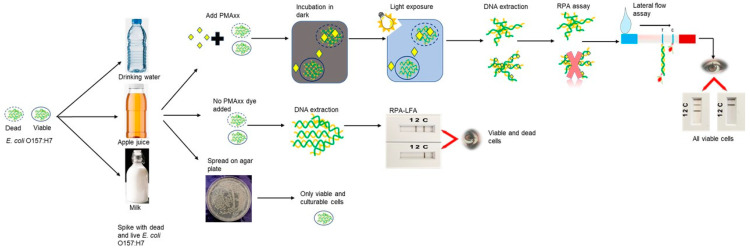
Schematic diagram of PMAxx-RPA-LFA for the detection of viable *Escherichia coli* O157:H7 in milk, apple juice, and drinking water.

The RPA reaction was conducted as per the manufacturer’s guidelines (TwistDX, San Diego, CA, USA). The lateral flow assay (LFA) was performed using the TwistAmp nfo Kit (TwistDX, San Diego, CA, USA). Primers and probes targeting the *rfbE* and *stx* genes were previously reported (Table S4) [34]. The RPA was carried out in a total reaction volume of 50 µL containing the following components: rehydration buffer (RB, 29.5 µL), nuclease free water (NFW, 11.2 µL), forward and reverse primers (2.1 µL), and nfo probe (0.6 µL). To reduce the risk of contamination, one master mix (MM) was prepared containing all components and stored at −20 °C until further use. In each, the reaction pellet MM (45.5 µL) and DNA template (2 µL) were added together with magnesium acetate (MgOAc, 2.5 µL). Tubes were briefly centrifuged and incubated at 39 °C for 15 min. Post amplification, the double labelled reporter amplicon was visualised using the PCRD device (Abingdon Health, UK) with minimum processing. The RPA amplicon (6 µL) was diluted with PCRD extraction buffer (84 µL from the kit), and an aliquot from the mixture (75 µL) was loaded onto the sample application area of the lateral flow (LF) cassette. Cassettes were observed for the presence of coloured control (C) and test (T) lines within 10 min (bands appear in 2–3 min). The presence of coloured bands in both the control and test lines indicated a positive result; a negative result was indicated by the presence of a coloured control line only. A schematic of the PMAxx-RPA-LFA process is shown in Figure 6.

Alongside, recombinase polymerase amplification-electrophoresis (RPA-EF) was conducted to confirm the size of the RPA amplicon using agarose gel electrophoresis (AGE). The TwistDX Basic RPA Kit was used for this; reactions (50 µL) were performed containing rehydration buffer (29.5 µL), nuclease free water (NFW, 11.2 µL), forward and reverse primers (2.4 µL, Table S3), DNA template (2 µL), and magnesium acetate (280 mM MgOAc, 2.5 µL). To avoid contamination and false positives, a master mix was prepared containing all constituents except for the template DNA and MgOAc. After dissolving the RPA pellet in the master mix (45.5 µL), DNA and MgOAc were added. Following brief centrifugation, tubes were incubated at 39 °C for 15 min. Post amplification, the RPA product was cleaned using a PCR Clean Up Kit (Bioline, London, UK), and the final product was observed on a 2% agarose gel [34].

For both RPA-LFA and RPA-EF, all primers and probes were commercially synthesised by Bioneer (Bioneer Corporation, Daejeon, Korea). For the non-template controls (NTC), uninoculated nutrient broth replaced bacterial culture for the PMAxx treatment.

### 4.5. Specificity of the PMAxx-Recombinase Polymerase Assay-Lateral Flow Assay (RPA-LFA)

The specificity of the primers and probes for the *rfbE* and *stx* genes was evaluated for PMAxx-RPA-LFA using 21 bacteria isolates including three *E. coli* O157:H7 isolates, 10 non target *E. coli* serotypes, and eight other non-target bacteria (Table 1). All bacterial isolates (heat-inactivated) were used at 10^8^ CFU mL^−1^ and treated with 100 µM PMAxx for 10 min followed by DNA extraction for RPA-LFA (see Section 4.2, Section 4.3 and Section 4.4).

### 4.6. Assessment of PMAxx-Recombinase Polymerase Assay for Pure E. coli O157:H7 Cultures and Spiked Commercial Samples

To evaluate the efficiency and sensitivity of the PMAxx-RPA assay, two experiments were performed with pure cultures. In Experiment 1, mixtures of different viable/dead cells ratios were used while keeping the total bacterial cell concentration constant (i.e., 10^8^ CFU mL^−1^). Early stationary phase bacterial cells (10^8^ CFU mL^−1^) were divided into two aliquots; one was used directly (viable), while the second was heat killed, as described in Section 4.2 and labelled as 100% dead cells. Viable and dead *E. coli* O157:H7 cells were then mixed in the ratio (vol/vol) 0:100, 30:70, 50:50, 70:30, and 100:0. In Experiment 2, viable cells at range of concentrations i.e., 10^2^, 10^3^, 10^4^, 10^5^, and 10^6^ CFU mL^−1^ and dead cells at 10^8^ CFU mL^−1^ were mixed. Pellets from the mixtures (1 mL) were collected and resuspended in 1X PBS (100 µL) and used for PMAxx treatment and DNA extraction as described in Section 4.3. Both RPA-LFA and RPA-EF assays were performed using both sets of primers (targeting *rfbE* and *stx* genes) as described in Section 4.4 including a set of controls reactions where PMAxx was not added.

For the commercial beverage samples, fresh full cream milk, apple juice, and bottled drinking water were purchased from a local supermarket in Melbourne, Australia. All samples were confirmed negative for *E. coli* O157:H7 by screening samples using plate culturing (on SMAC agar medium, 100 µL sample plated); all sorbitol negative isolates were further assessed using the serology based latex agglutination method with O157 antisera (Thermo Fisher Scientific, Waltham, MA, USA) following the manufacturer’s instructions [37]. Samples (milk, juice and water) were then inoculated with mixtures of viable and dead *E. coli* O157:H7 cells with different concentrations (10^2^:10^8^, 10^3^:10^8^, 10^4^:10^8^, 10^5^:10^8^, and 10^6^:10^8^ CFU mL^−1^). Cells were collected by centrifugation at 5000× *g* for 10 min followed by washing with 1X PBS and finally resuspended in 1X PBS (100 µL). All samples were treated with PMAxx (100 µM) followed by DNA extraction. The RPA-LFA assays were then carried out as described in Section 4.4, targeting only the *rfbE* gene. Control reactions for each sample type were also performed without the addition of PMAxx reagent. The lowest number of live *E. coli* O157:H7 cells detected using the PMAxx-RPA was defined as the LOD (CFU mL^−1^) for the assay.

### 4.7. Induction of Viable but Non Culturable (VBNC) E. coli O157:H7

*E. coli* O157:H7 was induced into the VBNC state using a previously described method [14]. In brief, bacterial cells (10^8^ CFU mL^−1^) were centrifuged and washed twice with 1X PBS; the cell pellet was resuspended in 7% NaCl (wt/vol) and incubated at 37 °C with agitation at 180 rpm until the desired VBNC state was induced. Daily, an aliquot (100 µL) was sampled and plated out on nutrient agar plates (in triplicate) to determine the culturable CFU mL^−1^ until the count decreased to 0 CFU mL^−1^. The state of VBNC cells was further confirmed using the LIVE/DEAD BacLight Bacterial Viability Kit, as described in Section 4.2.

### 4.8. Detection of Viable but Not Culturable (VBNC) E. coli O157:H7 in Artificially Contaminated Samples

For VBNC detection, all commercial samples were analysed for the absence of *E. coli* O157:H7 using the *Escherichia coli* O157 latex test (Thermo Fisher Scientific, Waltham, MA, USA) and plating on sorbitol MacConkey agar (SMAC) (Thermo Fisher Scientific, Waltham, MA, USA). Subsequently, the cell pellet from 1 mL of the induced VBNC *E. coli* O157:H7 was inoculated in each commercial sample (1 mL, milk, water, and apple juice) at a concentration ranging from 10^6^ to 10^2^ CFU mL^−1^. Following centrifugation at 5000× *g* for 10 min, the pellet was resuspended in 1X PBS buffer and used for PMAxx-RPA LFA, as described in Section 4.4. The lowest number of detected *E. coli* O157:H7 was determined as the LOD.

### 4.9. Effect of pH on the PMAxx-Recombinase Polymerase Assay-Lateral Flow Assay (RPA-LFA)

The impact of pH on the performance of the PMAxx-RPA-LFA assay was evaluated in terms of the detection of dead *E. coli* O157:H7. Water with a pH 6.5 was used as the substrate. Sodium hydroxide (NaOH, 1 M) and hydrochloric acid (HCl, 1 M) were used to adjust the pH of water prior to mixing with the cell pellets to reach pH 3 and pH 5 (acidic), pH 7 (neutral), and pH 11 (alkaline). The pellet from dead *E. coli* O157:H7 (2.3 × 10^8^ CFU mL^−1^) was mixed with 1 mL of each water sample (pH 3 to pH 11). The PMAxx treatment was applied, and DNA was extracted as described in Section 4.3 and used for RPA-LFA.

## 5. Conclusions

This study reports the application and optimisation of an RPA assay integrated with PMAxx treatment for the detection of only viable *E. coli* O157:H7 from the pure culture and commercial samples. The PMAxx-RPA-LFA assay represents a fast, simple, and reliable platform for differentiating viable and dead *E. coli* O157:H7 in milk, apple juice, and drinking water. In comparison to conventional culture methods, PMAxx-RPA-LFA not only elucidates dead cells, but also detects the VBNC bacterial population. The assay was found to be comparatively efficient when compared with the traditional culture methods and other molecular approaches. In addition, the problem of the overestimation of *E. coli* O157:H7 from food and beverage matrices is potentially addressed through the integration of PMAxx with RPA-LFA. Furthermore, RPA is an isothermal DNA amplification assay that can be completed using a simple heating instrument for incubation, which increases the applicability of the assay for field use (at point-of-care) or in resource-limited settings. Moreover, the PMAxx-RPA-LFA assay can be extended to other Gram-negative and Gram-positive food- and waterborne pathogens to supplement the ongoing efforts to increase food safety.

## Figures and Tables

**Table 1:** Specificity of PMAxx-RPA-LFA.

S. No.	Bacteria	Strain	Serotype	*rfbE* Gene		*stx* Gene	
				PMAxx-RPA-LFA	PMAxx-RPA-EF	PMAxx-RPA-LFA	PMAxx-RPA-EF
1.	*E. coli*	ATCC 43888	O157:H7	−	−	+	+
2.	*E. coli*	ATCC 43895	O157:H7	−	−	+	+
3.	*E. coli*	ATCC 8740	O157:H7	−	−	+	+
4.	*E. coli*	NCTC 8620	O26:H-	−	−	−	−
5.	*E. coli*	ATCC 25922	O6 biotype 1	−	−	−	−
6.	*E. coli*	NCTC 9001	O1:K1:H7	−	−	−	−
7.	*E. coli*	ATCC 23542	O129:H11	−	−	−	−
8.	*E. coli*	NCTC 11560		−	−	−	−
9.	*E. coli*	ATCC 35218		−	−	−	−
10.	*E. coli*	NCTC 8196	O103	−	−	−	−
11.	*E. coli*	ATCC 27325	W3110	−	−	−	−
12.	*E. coli*	ATCC 23716	K-12	−	−	−	−
13.	*E. coli*	Unknown	BL21	−	−	−	−
14.	*Staphylococcus aureus*	ATCC 6538	Unknown	−	−	−	−
15.	*Bacillus cereus*	ATCC 11778	Unknown	−	−	−	−
16.	*Klebsiella pneumonia*	ATCC 13883	Unknown	−	−	−	−
17.	*Pseudomonas aerouginosa*	ATCC 27853	Unknown	−	−	−	−
18.	*Shigella dysenteriae*	NCTC 4837	Unknown	−	−	−	−
19.	*Shigella flexneri*	Unknown	Unknown	−	−	−	−
20.	*Salmonella enteritidis*	ATCC 13076	Unknown	−	−	−	−
21.	*Listeria monocytogenes*	ATCC 19115	Unknown	−	−	−	−

+: positive; −: negative; PMAxx: propidium monazide; LFA: lateral flow assay; EF: electrophoresis; RPA: recombinase polymerase amplification.

## Data Availability

All data generated during this study are included in this published article and its supplementary document file.

## Supplementary Materials

The following supporting information can be downloaded at: https://www.mdpi.com/article/10.3390/foods11203207/s1. Figure S1: Raw data images for optimisation of the PMAxx-Recombinase Polymerase Assay (RPA) using PMAxx concentrations from 0 to 150 µM. (a) Lane M: molecular marker, Lane 2 to 7: RPA amplified DNA from dead *E. coli* O157:H7 using rfbE F1/R1. Lane 10–15: RPA amplified DNA from dead *E. coli* O157:H7 using stx F1/R1. [b&c] PMAxx treated live *E. coli* O157:H7 cells; (b) using rfbE F1/R1, (c) using stx F1/R1. Figure S2: Agarose gel electrophoresis of the PMAxx-RPA assay using different concentrations of *E. coli* O157:H7. (a) targeting *rfbE* gene (b) targeting *stx* gene. Lane M: molecular marker; Lane 1–8: contains dead *E. coli* O157:H7 at concentration 10^8^ CFU mL^−1^, 10^7^ CFU mL^−1^, 10^6^ CFU mL^−1^, 10^5^ CFU mL^−1^, 10^4^ CFU mL^−1^, 10^3^ CFU mL^−1^, 10^2^ CFU mL^−1^, and 10^1^ CFU mL^−1^. Lane 10–18: contains live *E. coli* O157:H7 at concentration 10^8^ CFU mL^−1^, 10^7^ CFU mL^−1^, 10^6^ CFU mL^−1^, 10^5^ CFU mL^−1^, 10^4^ CFU mL^−1^, 10^3^ CFU mL^−1^, 10^2^ CFU mL^−1^, and 10^1^ CFU mL^−1^. PMAxx treatment was given at 100 µM. Figure S3: Validation of PMAxx-Recombinase Polymerase Assay-Lateral Flow Assay (RPA-LFA) for pure *E. coli* O157:H7 with different concentrations of live and dead cells. (a) and (b) represent the RPA-LFA targeting *rfbE* gene; (c) and (d) represent the RPA-LFA targeting *stx* gene. [a & c]-LFA 1 to 5 PMAxx treated *E. coli* O157:H7 with 0:100, 30:70, 50:50, 70:30, 100:0 live and dead cell ratio. LFA 6 to 10 show control reactions without PMAxx treatment with the same ratio of viable and dead *E. coli* O157:H7. LFA 11 contains NTC. [b & d]-LFA 1 to 5 PMAxx treated *E. coli* O157:H7 with 10^2^:10^8^, 10^3^:10^8^, 10^4^:10^8^, 10^5^:10^8^, 10^6^:10^8^. LFA 6 to 10 show control reactions without PMAxx treatment with the same concentration of live and dead *E. coli* O157:H7 10^2^:10^8^, 10^3^:10^8^,10^4^:10^8^, 10^5^:10^8^, 10^6^:10^8^. LFA 11 ccontains NTC. Figure S4: Agarose gel electrophoresis of PMAxx-RPA for pure *E. coli* O157:H7 with different concentrations of viable and dead cells. Image (a) and (b) are representing the RPA assay targeting the *rfbE* gene; image (c) and (d) represent the RPA assay targeting the *stx* gene. [a & c]-lane 1 to 5: PMAxx treated *E. coli* O157:H7 with 0:100, 30:70, 50:50, 70:30, 100:0 viable and dead ratio. Lane 6 to 10 show control reactions without PMAxx treatment with the same ratio of viable and dead *E. coli* O157:H7. Lane 11 contains NTC. Lane M: 100 bp molecular marker. [b & d]-Lane 1 to 5 PMAxx treated *E. coli* O157:H7 with 10^2^:10^8^, 10^3^:10^8^, 10^4^:10^8^,10^5^:10^8^, 10^6^:10^8^. Lane 6 to 10 show control reactions without PMAxx treatment with the same concentrations of viable and dead *E. coli* O157:H7 10^2^:10^8^, 10^3^:10^8^, 10^4^:10^8^, 10^5^:10^8^, 10^6^:10^8^. Lane 11 contains NTC. Figure S5: Validation of PMAxx-Recombinase Polymerase Assay-Lateral Flow Assay (RPA-LFA) using the *rfbE* gene for commercial beverage samples spiked with different concentrations of live and dead cells, (a) milk samples; (b) water; (c) apple juice. LFA 1 to 6 PMAxx treated *E. coli* O157:H7 with 10^2^:10^8^, 10^3^:10^8^, 10^4^:10^8^, 10^5^:10^8^, 10^6^:10^8^, 0:10^8^, live:dead ratio, respectively. Lanes 7 to 12 show control reactions without PMAxx treatment with the same concentration of viable and dead *E. coli* O157:H7. Lane 13 shows NTC. Figure S6: Agarose gel electrophoresis of PMAxx-RPA for commercial beverage samples spiked with varying concentrations of viable and dead cells, (A) milk samples; (B) water; (C) apple juice. Lane M: 100 bp molecular marker, Lane 2 to 6 PMA treated *E. coli* O157:H7 with 10^2^:10^8^, 10^3^:10^8^, 10^4^:10^8^, 10^5^:10^8^, 10^6^:10^8^, 0:10^8^ live and dead. Lanes 7 to 12 show control reactions without PMA treatment with the same concentrations of viable and dead *E. coli* O157:H7 10^2^:10^8^, 10^3^:10^8^, 10^4^:10^8^, 10^5^:10^8^, 10^6^:10^8^. Lane 13 contains NTC. Figure S7: Effect of pH on PMAxx-RPA-LFA for the detection of dead *E. coli* O157:H7. LFA 1: pH 3; LFA 2: pH 5; LFA 3: pH 7; LFA 4: pH 11. Table S1: Summary of PMAxx-Recombinase Polymerase Assay-Lateral Flow Assay (RPA-LFA) results using different ratios and concentrations of Live:Dead *Escherichia coli* O157:H7 and viable but non-culturable cells (VBNC). Table S2: PMAxx-RPA-LFA outcomes in spiked commercial samples. Table S3: The unmodified primers for *E. coli* O157:H7 targeting *rfbE* and *stx* gene used for PMAxx-RPA-EF assay. Table S4: The modified primers and probes for *E. coli* O157:H7 targeting the *rfbE* and *stx* gene used for PMAxx-RPA-LFA.
