# Supplementary material for: Point-of-Care Lateral Flow Detection of Viable Escherichia coli O157:H7 Using an Improved Propidium Monoazide-Recombinase Polymerase Amplification Method
Source: Foods. 2022 Oct 14;11(20):3207. doi: 10.3390/foods11203207 (PMC9602316; doi:10.3390/foods11203207)
Supplement: Supplementary file 1 [file foods-11-03207-s001.zip › foods-1950625-Supplementary Document.pdf]

Supplementary Document

Point-of-Care Lateral Flow Detection of Viable *Escherichia coli* O157:H7 Using an Improved Propidium Monoazide-Recombinase Polymerase Amplification Method

Alka Rani [1,*], Charles Chinyere Dike [1], Nitin Mantri [2,3] and Andrew Ball [1]

[1] ARC Training Centre for the Transformation of Australia's Biosolids Resource, School of Science, RMIT University, Bundoora West, VIC 3083, Australia
[2] The Pangenomics Lab, School of Science, RMIT University, Melbourne, VIC 3083, Australia
[3] The UWA Institute of Agriculture, The University of Western Australia, Perth, WA 6009, Australia
* Correspondence: s3703623@student.rmit.edu.au; Tel.: +61-3-99-256594

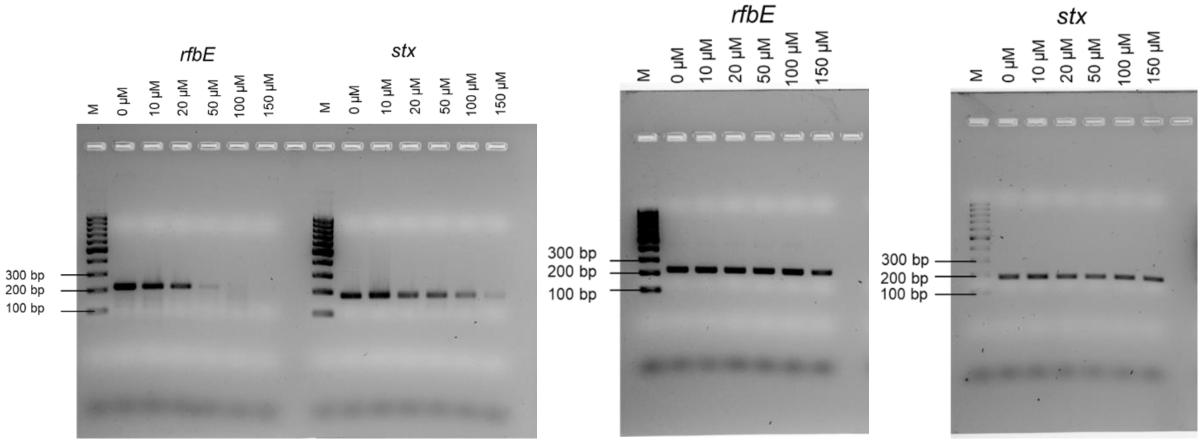

(a) (b) (c)

Figure S1: Raw data images for optimisation of the PMAxx-Recombinase Polymerase Assay (RPA) using PMAxx concentrations from 0 to 150 µM. (a) Lane M: molecular marker, Lane 2 to 7: RPA amplified DNA from dead *E. coli* O157:H7 using rfbE F1/R1. Lane 10-15: RPA amplified DNA from dead *E. coli* O157:H7 using stx F1/R1. [b&c] PMAxx treated live *E. coli* O157:H7 cells; (b) using rfbE F1/R1, (c) using stx F1/R1.

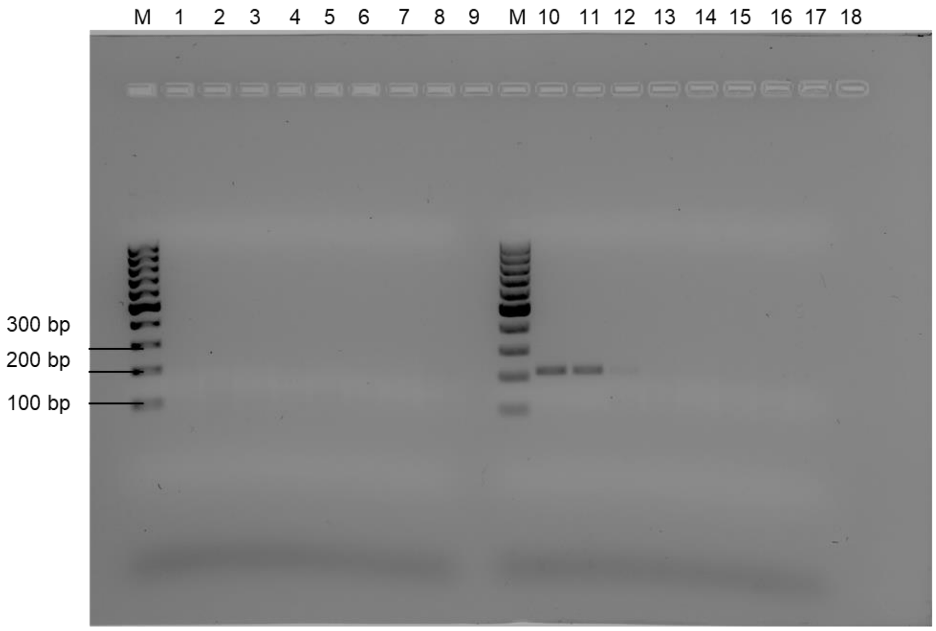

a

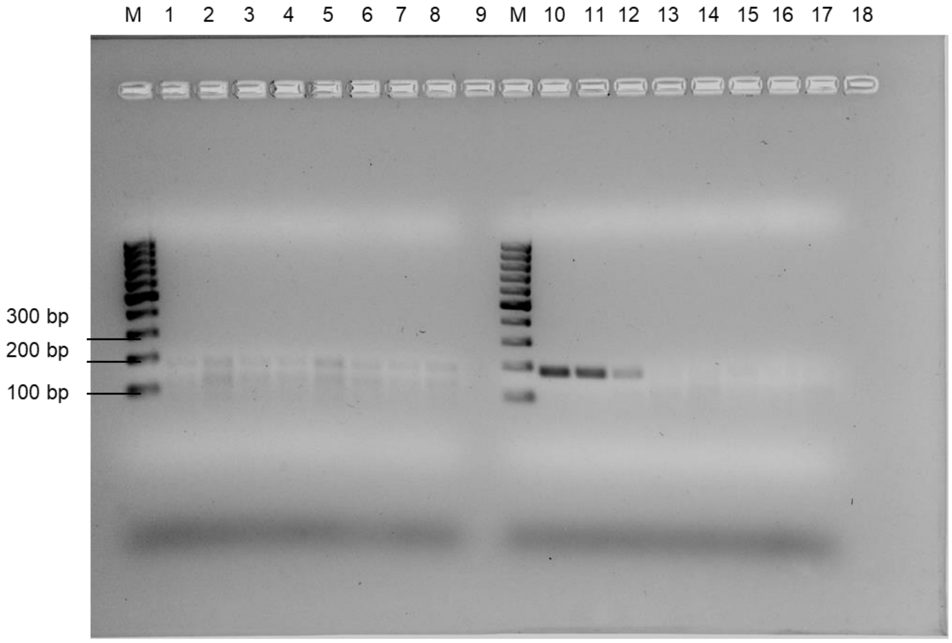

b

Figure S2: Agarose gel electrophoresis of the PMAxx-RPA assay using different concentrations of *E. coli* O157:H7. (a) targeting *rfbE* gene (b) targeting *stx* gene. Lane M: molecular marker; Lane 1-8: contains dead *E. coli* O157:H7 at concentration 10^8 CFU mL^{-1}, 10^7 CFU mL^{-1}, 10^6 CFU mL^{-1}, 10^5 CFU mL^{-1}, 10^4 CFU mL^{-1}, 10^3 CFU mL^{-1}, 10^2 CFU mL^{-1}, and 10^1 CFU mL^{-1}. Lane 10-18: contains live *E. coli* O157:H7 at concentration 10^8 CFU mL^{-1}, 10^7 CFU mL^{-1}, 10^6 CFU mL^{-1}, 10^5 CFU mL^{-1}, 10^4 CFU mL^{-1}, 10^3 CFU mL^{-1}, 10^2 CFU mL^{-1}, and 10^1 CFU mL^{-1}. PMAxx treatment was given at 100 µM.

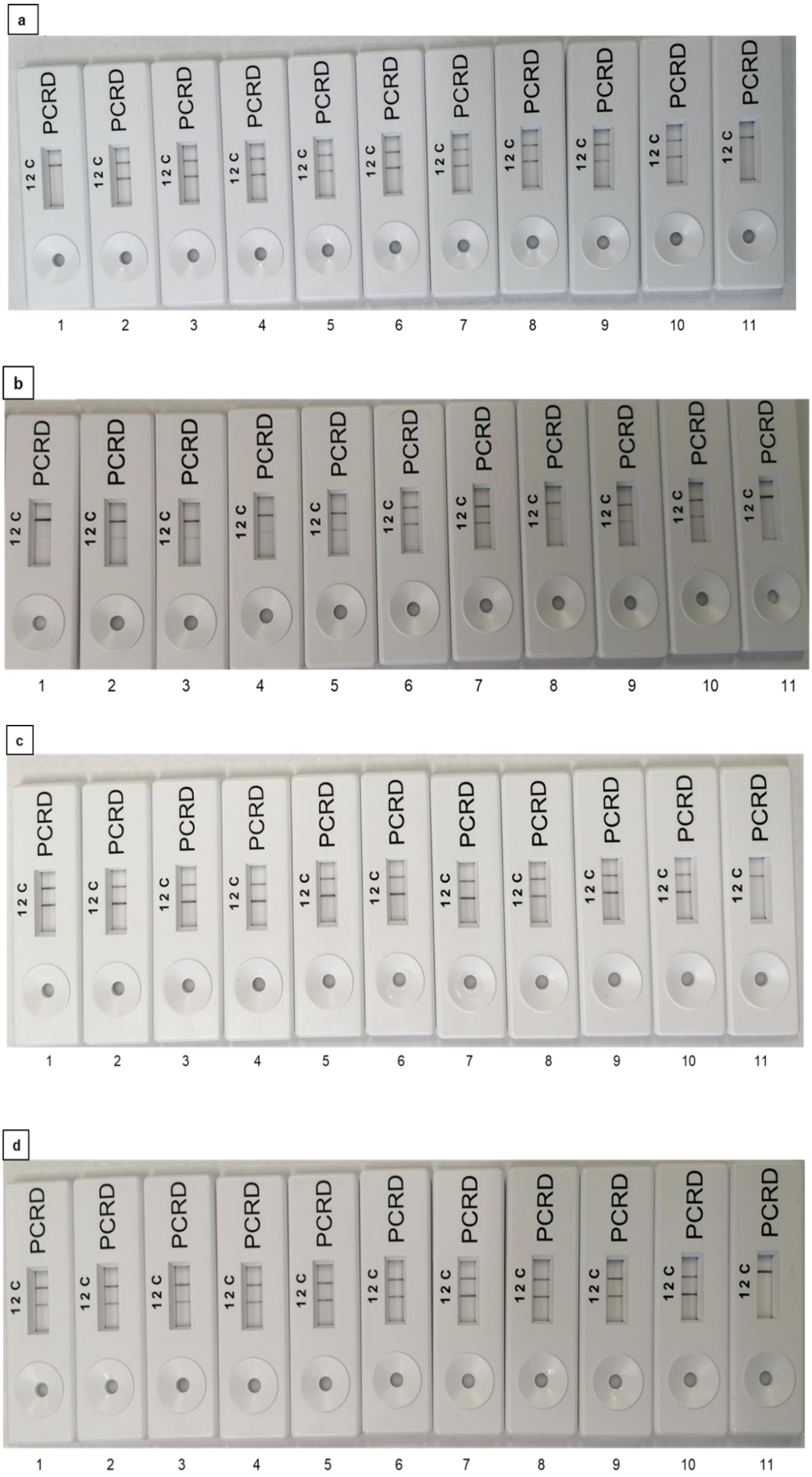
a
PCRD
12 C
1 2 3 4 5 6 7 8 9 10 11
b
PCRD
12 C
1 2 3 4 5 6 7 8 9 10 11
c
PCRD
12 C
1 2 3 4 5 6 7 8 9 10 11
d
PCRD
12 C
1 2 3 4 5 6 7 8 9 10 11

Figure S3: Validation of PMAxx- Recombinase Polymerase Assay-Lateral Flow Assay (RPA-LFA) for pure *E. coli* O157:H7 with different concentrations of live and dead cells. (a) and (b) represent the RPA-LFA targeting *rfbE* gene; (c) and (d) represent the RPA-LFA targeting *stx* gene. [a & c]- LFA 1 to 5 PMAxx treated *E. coli* O157:H7 with 0:100, 30:70, 50:50,70:30 ,100:0 live and dead cell ratio. LFA 6 to 10 show control reactions without PMAxx treatment with the same ratio of viable and dead *E. coli* O157:H7. LFA 11 contains NTC. [b & d]- LFA 1 to 5 PMAxx treated *E. coli* O157:H7 with 10^2:10^8, 10^3:10^8, 10^4:10^8,10^5:10^8 ,10^6:10^8. LFA 6 to 10 show control reactions without PMAxx treatment with the same concentration of live and dead *E. coli* O157:H7 10^2:10^8, 10^3:10^8,10^4:10^8, 10^5:10^8, 10^6:10^8. LFA 11 ccontains NTC.

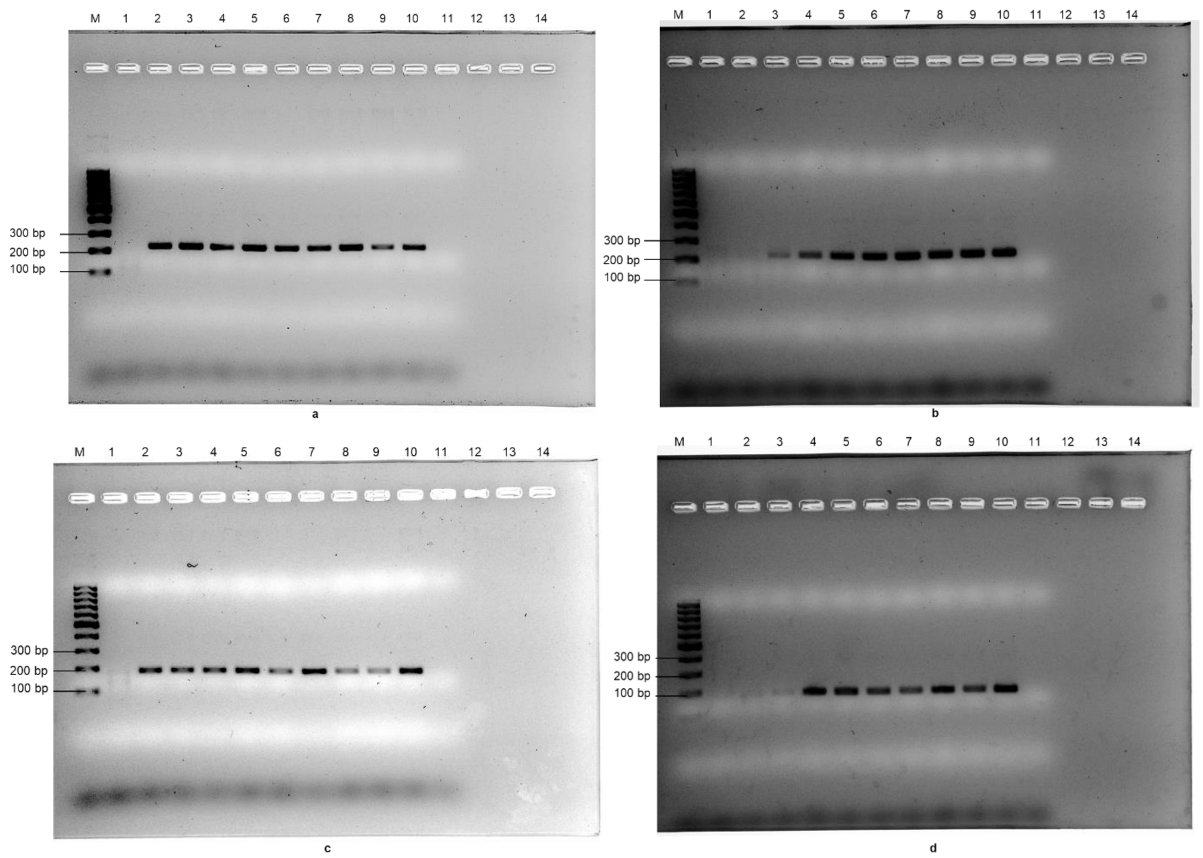

Figure S4: Agarose gel electrophoresis of PMAxx-RPA for pure *E. coli* O157:H7 with different concentrations of viable and dead cells. Image (a) and (b) are representing the RPA assay targeting the *rfbE* gene; image (c) and (d) represent the RPA assay targeting the *stx* gene. [a & c]- lane 1 to 5: PMAxx treated *E. coli* O157:H7 with 0:100, 30:70, 50:50,70:30 ,100:0 viable and dead ratio. Lane 6 to 10 show control reactions without PMAxx treatment with the same ratio of viable and dead *E. coli* O157:H7. Lane 11 contains NTC. Lane M: 100 bp molecular marker. [b & d]- Lane 1 to 5 PMAxx treated *E. coli* O157:H7 with 10^2:10^8, 10^3:10^8, 10^4:10^8,10^5:10^8 ,10^6:10^8. Lane 6 to 10 show control reactions without PMAxx treatment with the same concentrations of viable and dead *E. coli* O157:H7 10^2:10^8, 10^3:10^8,10^4:10^8, 10^5:10^8, 10^6:10^8. Lane 11 contains NTC.

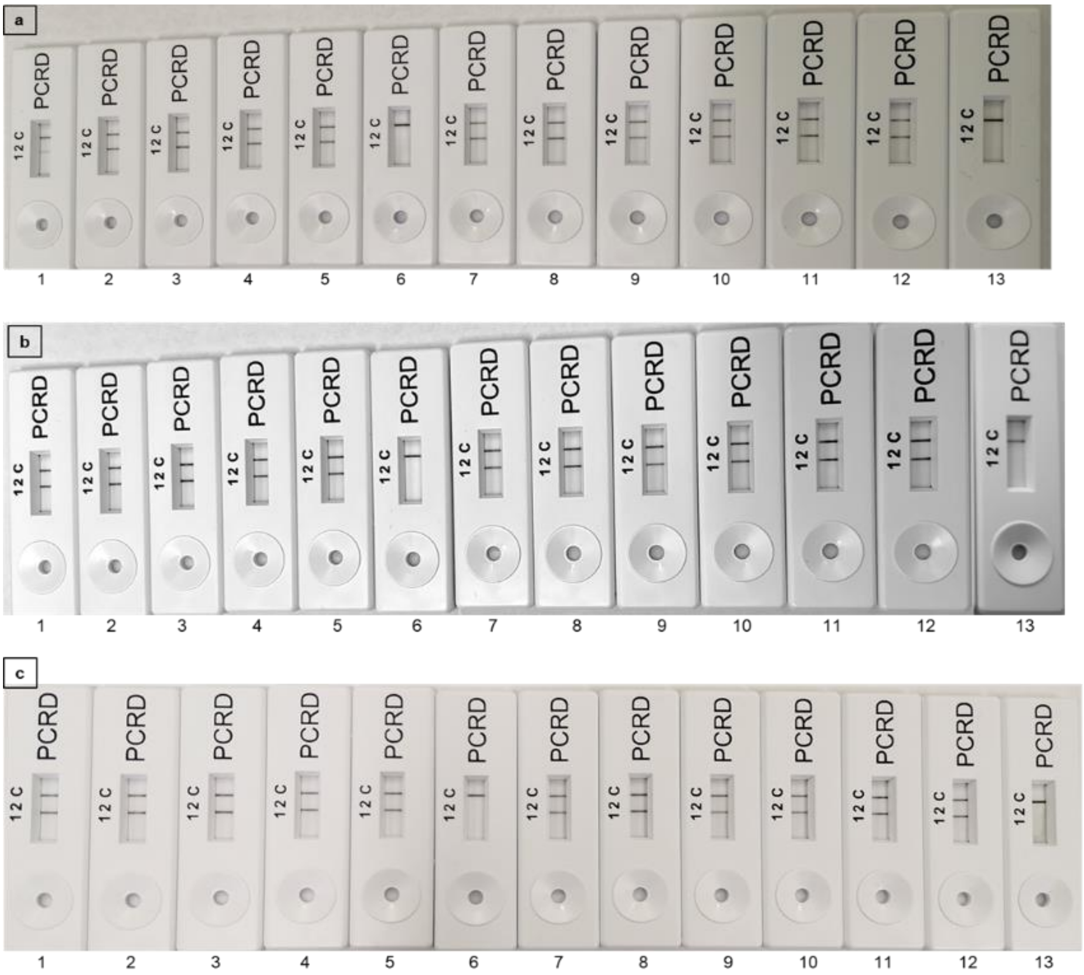

Figure S5: Validation of PMAxx- Recombinase Polymerase Assay-Lateral Flow Assay (RPA-LFA) using the *rfbE* gene for commercial beverage samples spiked with different concentrations of live and dead cells, (a) milk samples; (b) water; (c) apple juice. LFA 1 to 6 PMAxx treated *E. coli* O157:H7 with 10^2:10^8, 10^3:10^8, 10^4:10^8,10^5:10^8 ,10^6:10^8, 0:10^8, live:dead ratio, respectively. Lanes 7 to 12 show control reactions without PMAxx treatment with the same concentration of viable and dead *E. coli* O157:H7. Lane 13 shows NTC.

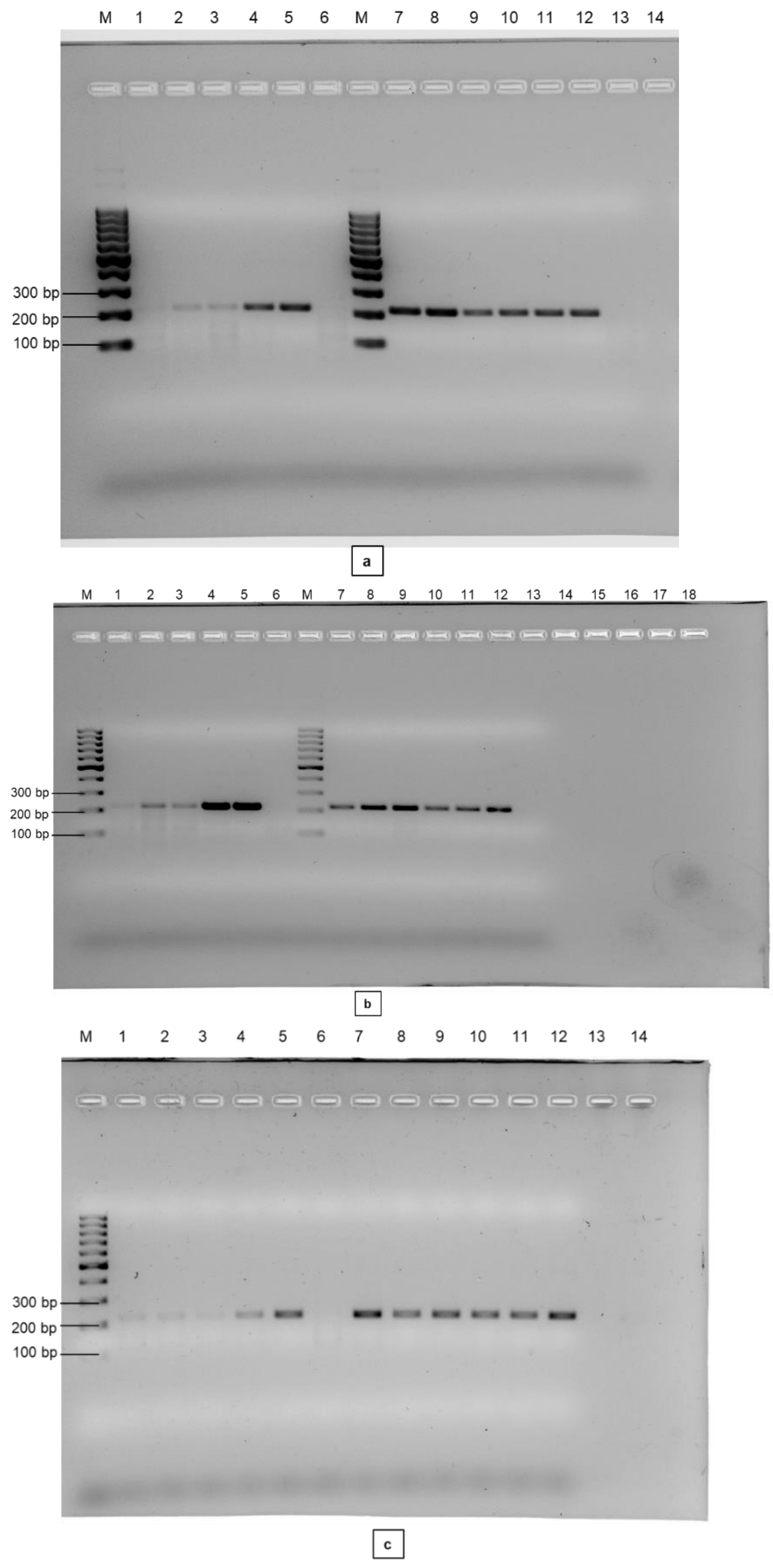

Figure S6: Agarose gel electrophoresis of PMAxx-RPA for commercial beverage samples spiked with varying concentrations of viable and dead cells, (A) milk samples; (B) water; (C) apple juice. Lane M: 100 bp molecular marker, Lane 2 to 6 PMA treated *E. coli* O157:H7 with 10^2:10^8, 10^3:10^8, 10^4:10^8,10^5:10^8 ,10^6:10^8, 0:10^8 live and dead. Lanes 7 to 12 show control reactions without PMA treatment with the same concentrations of viable and dead *E. coli* O157:H7 10^2:10^8, 10^3:10^8,10^4:10^8, 10^5:10^8, 10^6:10^8. Lane 13 contains NTC.

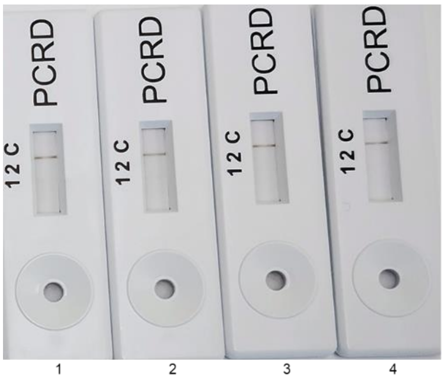

Figure S7: Effect of pH on PMAxx- RPA-LFA for the detection of dead *E. coli* O157:H7. LFA 1: pH 3; LFA 2: pH 5; LFA 3: pH 7; LFA 4: pH 11.

Table S1 Summary of PMAxx- Recombinase Polymerase Assay-Lateral Flow Assay (RPA-LFA) results using different ratios and concentrations of Live:Dead *Escherichia coli* O157:H7 and viable but non-culturable cells (VBNC)

a)

PMAxx-RPA-LFA / Live:Dead (CFU/mL)	Gene target *rfbE*	Gene target *stx*
(0: 10^8)	×	✓
(0: 10^7)	×	✓
(0: 10^6)	×	✓
(0: 10^5)	×	✓
(0: 10^4)	×	✓
(0: 10^3)	×	✓
(0: 10^2)	×	✓
(0: 10^1)	×	✓
(10^8:0)	✓	✓
NTC	×	×

b)

RPA-LFA / Live:Dead ratio (each 10^8 CFU/mL)	PMAxx treatment (*rfbE* primers)	No treatment (*rfbE* primers)	PMAxx treatment (*stx* primers)	No treatment (*stx* primers)
0:100	×	✓	✓	✓
30:70	✓	✓	✓	✓
50:50	✓	✓	✓	✓
70:30	✓	✓	✓	✓
100:0	✓	✓	✓	✓
NTC	×	×	×	×

c)

RPA-LFA / Live:Dead (CFU/mL)	PMAxx treatment (*rfbE* primers)	No treatment (*rfbE* primers)	PMAxx treatment (*stx* primers)	No treatment (*stx* primers)
(10^2:10^8)	✓	✓	✓	✓
(10^3:10^8)	✓	✓	✓	✓
(10^4:10^8)	✓	✓	✓	✓
(10^5:10^8)	✓	✓	✓	✓
(10^6:10^8)	✓	✓	✓	✓
NTC	×	×	×	×

×: No coloured band at test line (negative); ✓: coloured band at test line (positive); NTC: non-template control

Table S2 PMAxx-RPA-LFA outcomes in spiked commercial samples

a)

Live:Dead (CFU/mL) \ PMAxx-RPA-LFA	Milk	Water	Apple juice
(10^2:10^8)	✓	✓	✓
(10^3:10^8)	✓	✓	✓
(10^4:10^8)	✓	✓	✓
(10^5:10^8)	✓	✓	✓
(10^6:10^8)	✓	✓	✓
(0:10^8)	×	×	×
NTC	×	×	×

b)

VBNC (CFU/mL) \ PMAxx-RPA-LFA	Milk	Water	Apple juice	Pure culture
10^2	✓	✓	✓	✓
10^3	✓	✓	✓	✓
10^4	✓	✓	✓	✓
10^5	✓	✓	✓	✓
10^6	✓	✓	✓	✓
NTC	×	×	×	×

×: No colored band at test line (negative); ✓: colored band at test line (positive); NTC: non-template control; VBNC: viable but non culturable

Table S3 The unmodified primers for *E. coli* O157:H7 targeting *rfbE* and *stx* gene used for PMAxx-RPA-EF assay

Assay Type	Gene Target	Primers	Sequence (5'-3')	Primer length (bp)	Amplicon size (bp)	References
RPA-EF	*rfbE*	Eco rfbE F1	AGCTTTGTTAGCGTTAGGTATATCGGAAGGAGA	33	216	[34]
		Eco rfbE R1	ACATGGATGTCCGTATAAATGGACACACATAAT	33		
	stx	Eco stx2 F1	GTGGCCGGGTTCGTTAATACGGCAACAAATAC	32	179	
		Eco stx2 R1	TGAAACCAGTGAGTGACGACTGATTTGCATTC	32		

PMAxx- Propidium monoazide, RPA- recombinase polymerase amplification, EF- electrophoresis.

Table S4 The modified primers and probes for *E. coli* O157:H7 targeting the *rfbE* and *stx* gene used for PMAxx-RPA-LFA.

Assay Type	Gene target	Primers	Sequence (5’-3’)	Primer length (bp)	Amplicon size (bp)	References
RPA-LFA	*rfbE*	Eco rfbE F1	AGCTTTGTTAGCGTTAGGTATATCGGAAGGAGA	33	216	[34]
		Eco rfbE R1	**Bt**-ACATGGATGTCCGTATAAATGGACACACATAAT	33		
		Eco rfbE P1 probe	**5’-FAM-**GTTGATTCAGATAATGAAACTTGGCAAATGT-(**THFresidue**) TGTTAGTGACATAGAAC-**C3spacer-3’**	48		
RPA-LFA	*Stx*	Eco stx2 F1	5’-GTGGCCGGGTTCGTTAATACGGCAACAAATAC – 3’	32 nt	179	
		Eco stx2 R1	5’- Biotin-TGAAACCAGTGAGTGACGACTGATTTGCATTC – 3’	32 nt		
		Eco stx2 P1 probe	**5’- FAM-**AGTGCCCGGTGTGACAACGGTTTCCATGACAA**(THF residue)**GGACAGCAGTTATACCA-**C3spacer – 3’**	49		

PMAxx- Propidium monoazide, RPA- recombinase polymerase amplification, LFA- lateral flow assay

Rani, A., et al., *Evaluation and comparison of recombinase polymerase amplification coupled with lateral-flow bioassay for Escherichia coli O157: H7 detection using different genes.* Scientific reports, 2021. **11**(1): p. 1-12.
